# The Role of Emotional Intelligence and Frustration Intolerance in the Academic Performance of University Students: A Structural Equation Model

**DOI:** 10.3390/jintelligence13080101

**Published:** 2025-08-10

**Authors:** Ana María Ruiz-Ortega, María Pilar Berrios-Martos

**Affiliations:** 1 Department of Humanities, Faculty of Education and Social Sciences, Universidad Andrés Bello, Concepción 4180000, Chile; 2 Department of Social Psychology, Faculty of Psychology, University of Jaén, 23071 Jaén, Spain; pberrios@ujaen.es

**Keywords:** emotional intelligence, frustration intolerance, academic engagement, burnout, structural equation modeling, university students, academic performance

## Abstract

This study examines how emotional intelligence and frustration intolerance influence academic performance in university students, drawing on the Job Demands–Resources model—which frames academic success as a balance between psychological demands (such as frustration intolerance) and personal resources (like emotional intelligence)—and Self-Determination Theory, which explains how motivation and self-regulation contribute to adaptation and persistence in challenging contexts. A sample of 630 undergraduates across various disciplines completed validated measures of emotional intelligence, frustration intolerance, academic burnout, academic engagement, and grade point average. Structural equation modeling analyzed relationships among these variables. The results showed that emotional intelligence positively predicted academic performance both directly and indirectly by increasing engagement and reducing burnout. Conversely, frustration intolerance negatively affected academic performance through increased burnout and decreased engagement. The model explained 24 percent of the variance in academic performance. These findings indicate that academic achievement depends on managing the balance between psychological demands and personal resources. Frustration intolerance acts as a psychological demand increasing vulnerability to exhaustion and disengagement, while emotional intelligence serves as a personal resource supporting self-regulation, motivation, and persistence. This highlights the importance of fostering emotional skills and frustration tolerance in higher education to help students cope better with academic challenges and improve performance.

## 1. Introduction

Academic performance (AP) refers to the degree to which students achieve the learning goals established by their educational programs, commonly measured through exams, assignments, or overall grades (Ward et al. 1996). AP is increasingly recognized as a multifaceted construct involving not only knowledge acquisition but also skills and competencies shaped by cognitive, motivational, and emotional factors within diverse educational contexts (Privado et al. 2024). Understanding these factors is crucial, as academic achievement is a well-established predictor of individual outcomes such as career opportunities and socioeconomic mobility (Ng and Feldman 2009; Tebaldi et al. 2017), as well as broader societal development (Hanushek and Woessmann 2012; Liu et al. 2023).

Expected learning outcomes specify the knowledge, skills, and competences that students are expected to acquire upon completion of a learning process. They serve as key reference points that help align curriculum design, teaching strategies, and assessment practices (Cedefop 2022).

Grades, exams, and other assessment methods are widely recognized as valid indicators of both academic performance and expected learning outcomes, provided they are robustly designed and closely aligned with curricular goals. Employing a range of assessment types enhances the validity and reliability of conclusions regarding student achievement (Gupta 2023).

Recent research has increasingly highlighted the role of non-cognitive factors, such as emotional intelligence (EI) and frustration intolerance (FI), in shaping academic outcomes (Malanchini et al. 2024; Richardson et al. 2012). Although the relationship between academic emotions and learning outcomes has been widely studied (Tan et al. 2021), FI remains relatively underexplored despite its established links to emotional disengagement, academic burnout, and avoidance of challenging tasks, all of which can negatively impact AP (Feyzi Behnagh and Ferrari 2022). For example, students with high FI may abandon demanding assignments prematurely or experience heightened anxiety during exams, directly disrupting their academic progress. In contrast, higher EI—especially in the domain of self-regulation—has been shown to serve as a protective factor, helping students manage frustration, sustain engagement, and ultimately improve academic outcomes (Boekaerts and Pekrun 2015).

The impact of EI and FI on AP may vary according to gender, discipline, or cultural background (Halimi et al. 2020; Razumnikova and Mezentsev 2020). To better understand these dynamics, integrating perspectives from the Job Demands–Resources (JD-R) model (Bakker and Demerouti 2007) and Self-Determination Theory (SDT; Ryan and Deci 2000) is recommended, as they a provide a comprehensive framework for examining how EI operates as a personal resource and FI functions as a psychological demand within academic contexts.

Accordingly, this study clarifies how EI and FI jointly relate to AP in university students by integrating both constructs within a unified JD-R and SDT framework employing structural equation modeling (SEM). This novel approach offers fresh insights into the interplay between personal resources and psychological demands, with practical implications for higher education policy and intervention.

### 1.1. Conceptual Framework

As outlined in the JD-R model and SDT, academic outcomes depend on the balance between psychological demands—such as FI, which increases vulnerability to burnout and disengagement—and personal resources like EI, which facilitate adaptive coping, engagement, and resilience (Bao et al. 2024; Jagodics and Szabó 2023; Mérida-López et al. 2023). This integrated perspective underscores the importance of non-cognitive factors in academic success.

SDT complements this perspective by emphasizing that the satisfaction of basic psychological needs—autonomy, competence, and relatedness—underpins intrinsic motivation and engagement (Ryan and Deci 2000; Chen and Zhang 2022; Sabagh et al. 2021; Zupančič et al. 2024). EI supports these needs through emotional regulation and social adaptation, whereas FI disrupts self-regulation and impedes effective coping, ultimately increasing the risk of burnout and academic difficulties. For example, students with high EI are more likely to remain calm during exams, seek help proactively when facing difficult assignments, and recover quickly from setbacks, while those with low EI may become overwhelmed or disengaged in similar situations.

Despite the robust theoretical bases of JD-R and SDT, few studies have jointly examined EI and FI as predictors of AP in university students. Most prior research has addressed these constructs in isolation, limiting our understanding of their interplay and their combined impact on academic outcomes (Shi et al. 2021). Although some studies have explored JD-R and SDT together in occupational or school settings, their combined application to emotional and motivational factors in higher education remains rare, highlighting the novelty of the present approach.

Integrating JD-R and SDT enables a more comprehensive analysis of how emotional resources (EI) and demands (FI) interact with motivational processes to shape academic trajectories. This approach facilitates the examination of the joint contribution of these personal resources and psychological demands, alongside burnout and engagement, as key constructs highlighted by both frameworks.

Recent studies show that the JD-R model has been adapted to educational contexts, incorporating person–situation and multilevel approaches to better understand student adjustment and academic performance (Bakker et al. 2023). Similarly, meta-analyses confirm that SDT-based interventions reliably enhance student motivation, autonomy, competence, and engagement across diverse settings (Wang et al. 2024). Empirical work also highlights the relevance of SDT in fostering engagement, especially in digital and self-directed learning environments (Yang et al. 2025). However, research exploring the combined effects of EI and FI within these frameworks in university students remains limited, indicating a relevant gap addressed by this study.

### 1.2. Emotional Intelligence and Frustration Intolerance: Key Factors in Academic Success

EI is a crucial non-cognitive factor in AP, defined as the ability to perceive, understand, and regulate emotions in oneself and others (Mayer and Salovey 1997). Higher EI contributes to emotional stability, supports adaptation to academic challenges, and facilitates effective interpersonal functioning in academic contexts (Sandoval and Castro 2016; Quílez-Robres et al. 2023). Empirical research supports the link between EI and academic functioning, showing that students with higher EI report better academic results, greater academic engagement, and more effective responses to stress (MacCann et al. 2019; Perera and DiGiacomo 2013; Thomas and Allen 2021). In line with the JD-R model, EI can be conceptualized as a personal resource that helps students manage emotional demands and maintain high levels of engagement (Bakker and Demerouti 2007). From the perspective of SDT, EI also facilitates the satisfaction of basic psychological needs (autonomy, competence, relatedness), supporting motivation and academic persistence (Ryan and Deci 2000).

In contrast, FI refers to the inability to tolerate adverse or frustrating situations, often accompanied by exaggerated emotional reactions and maladaptive coping (Ellis 1962). FI is driven by irrational beliefs, such as “I must always succeed,” which distort students’ perception of academic challenges, increasing perceived threat and emotional vulnerability. Within the JD-R framework, FI functions as a psychological demand that drains emotional resources, increases burnout risk, and undermines academic engagement.

FI has been empirically linked to negative academic behaviors such as procrastination (Harrington 2005a) and lower academic achievement (Shi et al. 2021). Moreover, students with low frustration tolerance are more likely to avoid difficult academic tasks and disengage when outcomes do not meet expectations (Wu et al. 2023).

Emerging research suggests that EI and FI are inversely related, with students with higher EI exhibiting greater frustration tolerance and more constructive coping with academic demands (Kumari and Gupta 2015). This dynamic interaction indicates that interventions designed to enhance EI may also reduce the negative impact of FI, promoting both emotional resilience and academic performance.

### 1.3. Burnout and Academic Engagement: Two Sides of the Same Coin

Recent research indicates that burnout and engagement are distinct but interconnected states that can co-occur and fluctuate depending on students’ emotional and motivational regulation, rather than being strict opposites (Cano et al. 2024; Salanova and Schaufeli 2004).

Academic burnout, marked by emotional exhaustion, cynicism, and reduced efficacy, typically results from prolonged academic stress and excessive demands (Schaufeli et al. 2002; Madigan and Curran 2021; Xie et al. 2019). This state can lead to emotional detachment, a sense of low accomplishment, and declining motivation, all of which increase the risk of poor AP and dropout (Marôco et al. 2020; Paloș et al. 2019; Reyes-de-Cózar et al. 2023).

In contrast, engagement is a positive state characterized by vigor, dedication, and absorption in learning (Schaufeli and Bakker 2004; Salanova et al. 2010). Students who are engaged tend to achieve better results, persist longer, and report greater well-being (Zhoc et al. 2020; Maguire et al. 2017), as they invest effort, adapt to challenges, and remain committed to their studies.

For instance, a student might actively participate in class discussions and complete assignments on time (high engagement), yet still feel emotionally drained at the end of the week (burnout). Conversely, another student may feel indifferent and withdraw from group work (low engagement), even if not experiencing marked exhaustion (low burnout) (Salmela-Aro and Read 2017).

EI supports engagement and protects against burnout through effective emotional regulation (Iuga and David 2024), while FI increases the risk of both burnout and disengagement due to poor coping strategies and heightened emotional reactivity (Buzzai et al. 2021; Delgado-Floody et al. 2020; Potard and Landais 2021; Zhang and Jiang 2023).

### 1.4. The Current Study

This study addresses a key gap in the literature by jointly examining EI, FI, academic engagement, and burnout as predictors of AP. SEM was chosen because it enables simultaneous estimation of direct and indirect relationships among multiple variables, providing a comprehensive analysis of the complex pathways linking emotional and motivational factors to academic outcomes.

By integrating the JD-R model and SDT, this study explores how emotional resources (EI) and demands (FI) interact to influence academic outcomes through motivational and affective mechanisms, recognizing that academic success depends not only on cognitive abilities, but also on students’ capacity to regulate emotions, tolerate frustration, and sustain engagement in the face of challenges.

**Figure 1:**
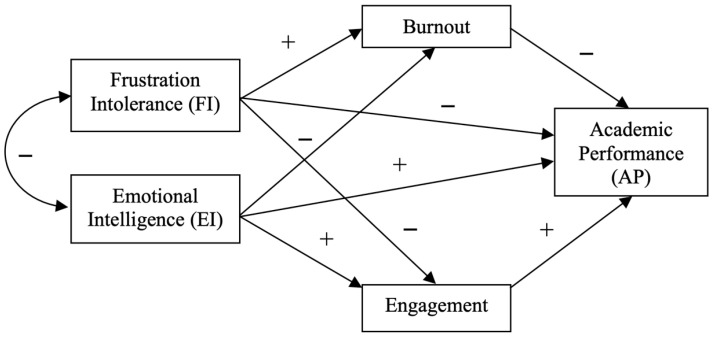
Hypothesized model.

Accordingly, the following objectives and hypotheses are proposed (see Figure 1):

**Objectives** **1.** *To verify whether EI positively influences AP, both directly and indirectly, through increased engagement and decreased burnout.*

**Hypothesis** **1.** *EI will be positively associated with AP, both directly and indirectly via higher engagement and lower burnout.*

**Objectives** **2.** *To analyze whether FI negatively influences AP, both directly and indirectly, through increased burnout and decreased engagement.*

**Hypothesis** **2.** *FI will be negatively associated with AP, both directly and indirectly via higher burnout and lower engagement.*

**Objectives** **3.** *To examine whether EI contributes to lower FI and whether FI, in turn, influences EI, reflecting a potential bidirectional relationship.*

**Hypothesis** **3.** *EI and FI will be negatively associated, supporting a bidirectional relationship between these two variables.*

## 2. Materials and Methods

### 2.1. Participants, Design, and Procedure

A total of 630 undergraduate students from Universidad Andrés Bello, Concepción (Chile), participated in this cross-sectional, correlational study. The university was selected for its disciplinary diversity and large student body, providing a broad representation of academic disciplines in the region. The sample included 403 women (64%) and 227 men (36%), with ages ranging from 18 to 37 years (M = 20.76, SD = 2.42). Most participants were within the typical undergraduate age range of 18 to 25 years, with a small proportion being older, minimizing age-related heterogeneity. Students were predominantly enrolled in health sciences (68.4%, n = 431), followed by technical/experimental disciplines (18.9%, n = 119) and social sciences (12.5%, n = 79). Regarding academic year, 190 (30.2%) were first-year, 340 (54.0%) second-year, 40 (6.3%) third-year, 45 (7.1%) fourth-year, and 15 (2.4%) fifth-year students.

The inclusion criteria were as follows: (a) being a regular undergraduate student, defined by full-time enrollment in one’s degree program during the data collection period, (b) being aged 18 or older, and (c) providing informed consent and authorization to access academic records. No exclusion criteria were applied beyond incomplete survey responses. Recruitment was open to all undergraduate students, conducted via institutional communication and classroom announcements. Participation was voluntary, anonymous, and without incentives.

Although participation was anonymous, students provided explicit consent for academic grade retrieval from official university records, linked in a de-identified manner using unique codes to ensure confidentiality. The predominance of health sciences students and early academic years is acknowledged as a source of potential sampling bias, which may limit generalizability to other disciplines and more advanced students.

### 2.2. Measures

- *Sociodemographic data*. Participants provided information on age, gender, academic year, and degree program through a brief questionnaire.
- *The Trait Meta-Mood Scale* (TMMS-24; Fernández-Berrocal et al. 2004; validated in Chile by Espinoza et al. 2015), developed by Salovey et al. (1995), was used to assess EI. This instrument consists of 24 items rated on a 5-point Likert scale (1 = never, 5 = very often) and evaluates three dimensions: attention to feelings, emotional clarity, and emotional repair. Each dimension is measured by eight items. Higher scores indicate greater perceived EI. In the present study, we used a Cronbach’s alpha of 0.93. The subscales also showed high reliability: attention (α = 0.91), clarity (α = 0.92), and repair (α = 0.90).
- *The Frustration Discomfort Scale* (FDS; Harrington 2005b; validated in Chile by Ruiz-Ortega et al. 2023) was used to measure FI. The scale includes 28 items, divided into four subscales: discomfort intolerance, entitlement, emotional intolerance, and achievement. Responses are given on a 5-point Likert scale (1 = not at all, 5 = very much). Higher scores reflect greater intolerance to frustration. The total scale showed a Cronbach’s alpha of 0.95 in this sample (discomfort intolerance: 0.85; entitlement: 0.86; emotional intolerance: 0.87; achievement: 0.82).
- *The Maslach Burnout Inventory Student Survey* (MBI-SS; Maslach et al. 1996; validated in Chile by Pérez et al. 2012). This 22-item instrument uses a 7-point Likert scale (0 = never, 6 = always) and measures three dimensions: exhaustion, cynicism, and academic efficacy. Higher scores in exhaustion and cynicism, and lower scores in efficacy, indicate greater burnout. The total scale showed a Cronbach’s alpha of 0.76 in this study (exhaustion: 0.91; cynicism: 0.72; efficacy: 0.79).
- *The Utrecht Work Engagement Scale Students* (UWES-S-9; Schaufeli et al. 2006) was used to assess academic engagement. This scale consists of 9 items rated on a 7-point Likert scale (0 = never, 6 = always), measuring vigor, dedication, and absorption. Higher scores indicate higher engagement. The total scale showed a Cronbach’s alpha of 0.94 in this study (Vigor: 0.86; Absorption: 0.89; Dedication: 0.88).
- *Academic performance.* GPA from the previous semester was used as the indicator of academic achievement, obtained from official university records, using Chilean standard grading (1 to 7) untransformed for transparency. The GPA represents the mean of all final course grades from the prior semester, reflective of each program’s assessment system, and includes a range of evaluation types (e.g., written and oral exams, assignments, practical work, and participation).

### 2.3. Data Analysis

The data were analyzed using Jamovi (version 2.3.21) and RStudio (version 2023.06.0). Prior to the main analyses, the dataset was examined for data entry accuracy, missing values (less than 2%), outliers, and adherence to assumptions. Missing data were minimal and handled using listwise deletion, under the assumption that data were missing completely at random (MCAR). Descriptive statistics and reliability indices were calculated for all variables. Preliminary analyses detected no significant differences by gender, age, or academic discipline.

Due to some deviations from multivariate normality, robust maximum likelihood estimation (MLR) was employed for confirmatory factor analyses (CFAs) and SEM to obtain reliable parameter estimates. The SEM tested the joint effects of EI and FI on AP, considering engagement and burnout as key motivational and emotional processes within the frameworks of the JD-R model and SDT.

Model fit was evaluated with chi-square (χ^2^), Comparative Fit Index (CFI), Tucker–Lewis Index (TLI), Root Mean Square Error of Approximation (RMSEA), and Standardized Root Mean Square Residual (SRMR), using commonly accepted cutoff criteria. Bias-corrected bootstrapping with 5000 resamples was performed to assess the significance and robustness of indirect effects. All statistical tests were two-tailed, with significance set at *p* < .05.

## 3. Results

### 3.1. Correlation Analysis

Table 1 displays the means, standard deviations, reliability indices, Cronbach’s alpha reliability coefficients (on the diagonal), and Pearson correlations for all study variables and their main dimensions. EI, particularly the clarity and repair dimensions, showed moderate positive correlations with AP, while emotional attention was not significantly associated. All dimensions of academic engagement (vigor, dedication, and absorption) showed positive associations with AP, with dedication displaying the strongest relationship. Conversely, burnout dimensions (emotional exhaustion, cynicism, and reduced academic efficacy) correlated negatively with AP, especially exhaustion and cynicism. FI, notably emotional intolerance, also showed negative correlations with AP, indicating that higher FI relates to poorer outcomes. 

EI was positively associated with engagement and negatively associated with burnout and FI. The engagement dimensions were negatively correlated with burnout and FI, while burnout and FI were positively correlated. These patterns emphasize the significant roles of emotional regulation, academic engagement, and effective management of burnout and FI for academic success among university students. For complete statistics, see Table 1.

### 3.2. Confirmatory Factor Analysis

CFA was conducted for each instrument using the MLR, due to the non-normality and large sample size (N > 500). Pre-CFA data screening indicated that most variables exceeded recommended skewness (≤3) and kurtosis (≤10) thresholds (Kline 2016). Multicollinearity was assessed via variance inflation factor (VIF < 5) revealing no concerns, and no influential outliers were detected.

The CFA results supported the expected factorial structure for all instruments. Items with standardized factor loadings below 0.5 were removed to ensure construct validity and model parsimony. The retained item loadings ranged from 0.53 to 0.85. Reliability analyses yielded acceptable internal consistency, with Cronbach’s alpha and Omega coefficients exceeding 0.7 for all constructs (see Table 1), confirming dimensionality and measurement reliability. The analyses followed current guidelines for ensuring measurement validity and addressing potential method bias (Steenkamp and Maydeu-Olivares 2021).

### 3.3. Model Fit Assessment

The measurement model, incorporating latent variables for EI, FI, burnout, and engagement, exhibited good fit to the data (see Table 2). The ratio of chi-square to degrees of freedom (χ^2^/df = 2.93) was below the recommended cutoff of 5 (Kline 2016), indicating acceptable model fit. The Comparative Fit Index (CFI = 0.969) and Tucker–Lewis Index (TLI = 0.958) exceeded the conventional threshold of 0.95 (Hu and Bentler 1999), suggesting excellent model fit. The Root Mean Square Error of Approximation (RMSEA = 0.055) was below 0.06 (Browne and Cudeck 1992), and the Standardized Root Mean Square Residual (SRMR = 0.044) was well under the recommended 0.08 threshold (Hu and Bentler 1999), further confirming model adequacy.

To verify the measurement model’s robustness, alternative nested models were tested. Models with constrained factor loadings or excluding indirect paths demonstrated significantly poorer fit (CFI < 0.90, RMSEA > 0.08), supporting the appropriateness of the hypothesized measurement model.

### 3.4. The Structural Equation Model

**Figure 2:**
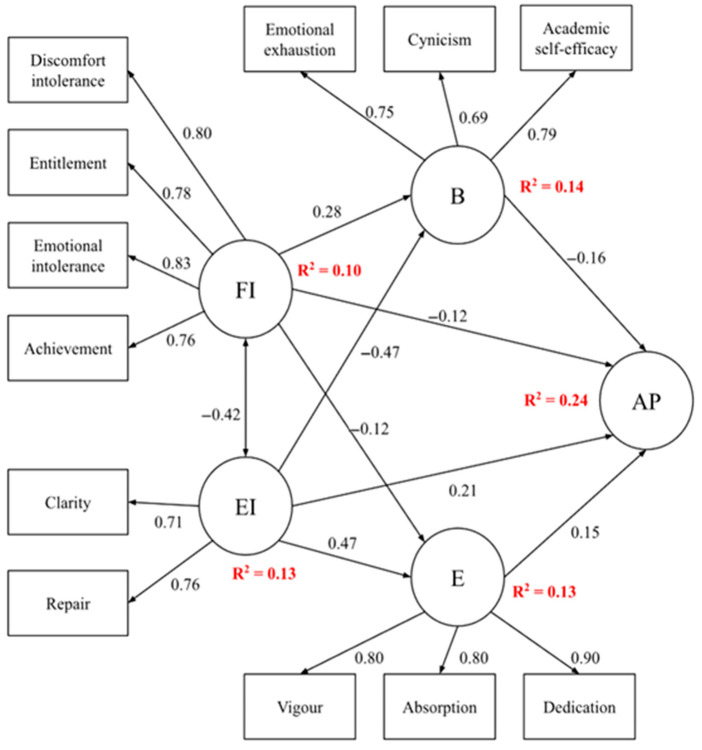
Path model.

Table 3 and Figure 2 present the standardized direct and indirect effects estimated in the SEM. EI showed a significant direct positive effect on AP (β = 0.21, *p* < .001), alongside indirect effects through increased engagement (β = 0.13, *p* < .001) and reduced burnout (β = −0.07, *p* < .001). Conversely, FI exhibited a significant direct negative effect on AP (β = −0.12, *p* < .01), as well as indirect effects via engagement (β = −0.06, *p* < .01) and burnout (β = −0.03, *p* < .01). Engagement was positively associated with AP (β = 0.31, *p* < .001), while burnout was negatively associated (β = −0.23, *p* < .001). All coefficients are standardized and accompanied by standard errors and *p*-values.

The SEM was designed to assess how EI and FI influence AP directly and indirectly through motivational (engagement) and emotional (burnout) processes, within the theorical frameworks of the JD-R and SDT. This dual-pathway approach clarifies the interplay between psychological resources and demands in academic achievement. Model fit indices showed excellent fit (CFI = 0.969, TLI = 0.958, RMSEA = 0.055, SRMR = 0.044), supporting the robustness of the model.

Interpretation of the standardized path coefficients reveals that EI is a robust predictor of both increased engagement (β = 0.472, *p* < .001) and decreased burnout (β = −0.472, *p* < .001), confirming its dual role as a personal resource that promotes academic motivation and protects students from exhaustion. These positive effects of EI are observed not only in its direct influence on AP, but also indirectly via higher engagement and lower burnout. In contrast, FI operates as a psychological demand: it significantly elevates burnout (β = 0.276, *p* < .001) and reduces engagement (β = −0.121, *p* = .010), and this undermining impact is transmitted both directly (β = −0.123, *p* = .012) and indirectly—through both increased burnout and reduced engagement—on AP. Engagement itself has a direct positive effect on AP (β = 0.154, *p* < .001), while burnout shows a negative direct effect (β = −0.157, *p* = .008), reinforcing their centrality as explanatory mechanisms within the model.

The model explains 24% of the variance in AP (R^2^ = 0.24), a moderate effect size consistent with the existing literature on academic achievement. Bias-corrected confidence intervals based on 5000 resamples validated the significance and stability of all direct and indirect effects, enhancing confidence in the results. Alternative models excluding indirect pathways (focusing on direct effects only) or restricting bidirectional paths showed significantly poorer fit indices (CFI < 0.90, RMSEA > 0.08), providing empirical justification for maintaining the full hypothesized model encompassing both direct and indirect effects.

All SEM assumptions were tested and met. Robust MLR addressed non-normality, and the large sample size (N = 630) ensured the stability of parameter estimates. No estimation or convergence issues arose. Nonetheless, the cross-sectional design restricts causal inference, and the use of self-report measures could introduce shared method variance, so these limitations should be considered.

In summary, the SEM results provide strong support for the hypothesized direct and indirect influences of EI and FI on AP via engagement and burnout. This integrated model highlights the dynamic interplay of psychological resources and demands shaping motivational and emotional processes that ultimately affect academic success in university students.

## 4. Discussion

This study confirms the central role of EI as a personal resource and FI as a psychological demand in AP, extending prior research by jointly modeling these constructs within a unified JD-R and SDT framework for the first time. Importantly, by integrating EI and FI simultaneously, our findings provide new evidence on how these factors interactively shape students’ academic adaptation—a perspective not addressed in previous research. These results confirm the international relevance and value of this integrated framework, understanding AP across diverse educational systems.

Consistent with our first hypothesis (H1), EI was positively associated with AP, both directly and indirectly through increased engagement and reduced burnout. This aligns with previous research demonstrating that students with higher EI are more capable of managing academic stress, sustain motivation, and achieve superior academic results (MacCann et al. 2019; Perera and DiGiacomo 2013). The positive indirect effect of EI via engagement and burnout underscores the importance of emotional regulation skills not only for academic achievement, but also for psychological well-being and resilience (Salanova et al. 2010; Spedding et al. 2017). Our findings also reveal that EI enhances engagement in academic tasks—a well-established predictor of AP (Maguire et al. 2017; Thomas and Allen 2021)—and that students with higher EI are more likely to remain engaged and motivated despite academic challenges (Upadyaya and Salmela-Aro 2015). Additionally, EI was negatively correlated with burnout, consistent with research showing that those with higher EI experience less emotional exhaustion (Molero-Jurado et al. 2021; Loi and Pryce 2022). This reinforces the argument that EI serves as a protective factor, as demonstrated by Salanova et al. (2010) and Bresó et al. (2011), where students with higher vigor and dedication achieved better AP.

Regarding our second hypothesis (H2), FI was found to negatively impact AP, both directly and indirectly, by promoting burnout and reducing engagement. This is consistent with research showing that students with greater FI experience higher levels of emotional exhaustion and disengagement, which in turn undermines their AP (Feyzi Behnagh and Ferrari 2022; Harrington 2005b). The indirect pathways observed in our model highlight the role of maladaptive emotional responses and motivational withdrawal as mechanisms linking FI to poorer academic outcomes. Previous studies have also shown that low frustration tolerance is associated with higher stress, lower well-being, and a greater risk of burnout and academic difficulties (Bakare et al. 2019; Sabagh et al. 2021; Srivastava 2024).

Importantly, our results also support the third hypothesis (H3), revealing a significant inverse relationship between EI and FI, which further strengthens the novelty of jointly modeling these constructs. This aligns with previous studies reporting that individuals with higher EI tend to exhibit greater frustration tolerance. For instance, Kumari and Gupta (2015) observed a positive correlation (r = 0.17, *p* < 0.01) between EI and frustration tolerance, concluding that students with higher EI are better equipped to handle frustration. This relationship is practically relevant in educational settings, as it implies that fostering EI may simultaneously strengthen students’ ability to cope with academic setbacks, promoting both emotional resilience and sustained engagement, even in those with higher FI.

Our combined model thus advances the literature by demonstrating that EI and FI do not simply exert independent influences, but can also interact such that high EI may compensate for, or buffer, the negative consequences of high FI. This integrative perspective answers the ‘so what’ question: developing students’ EI could both enhance their engagement and directly mitigate the academic vulnerabilities related to FI.

The SEM explained 24% of the variance in AP, which is considered moderate and aligns with previous studies using similar models in higher education contexts (MacCann et al. 2019; Salanova et al. 2010; Fiorilli et al. 2017). This demonstrates that emotional and motivational variables, while not the sole determinants, play a substantial role in academic achievement and should be considered alongside cognitive and contextual factors (Martínez-Rodríguez and Ferreira 2025; Sutil-Rodríguez et al. 2024; Wang and Raman 2025).

### 4.1. Theoretical Elaboration

In addition to the empirical results, our findings contribute to theoretical understanding by reinforcing the JD-R model and SDT in academic contexts involving EI, FI, engagement, burnout, and AP. Specifically, our data suggest that EI operates not only as a direct resource, but also as a buffer against the negative effects of FI, sustaining engagement and reducing burnout even when students are exposed to high levels of frustration or academic challenges. This is a critical, practical insight for educational psychology and aligns with the notion of resource–demand interplay in the JD-R framework.

From a SDT perspective, EI may facilitate the satisfaction of basic psychological needs (autonomy, competence, relatedness), essential for motivation and persistence. The observed inverse EI–FI relationship supports self-regulation and resilience models, showing that increasing EI may enhance frustration tolerance. Conceptualizing FI as a psychological demand clarifies its role in eliciting maladaptive emotional and motivational responses leading to burnout, consistent with stress and coping theories. The dynamic interplay between EI as a resource and FI as a demand in the JD-R framework provides a comprehensive, integrative explanation of student adaptation, engagement, and performance.

These theoretical insights are supported by a growing body of international research, underscoring the broad applicability and relevance of integrating these frameworks to understand academic achievement across diverse educational systems (Bakker and Demerouti 2007; Ryan and Deci 2000; MacCann et al. 2019).

These insights underscore the importance of addressing both personal resources and psychological demands in interventions targeting student well-being and achievement, advancing educational psychology models by highlighting emotional competencies and frustration tolerance as key interacting determinants of academic success.

### 4.2. Implications for Practice

These findings offer important practical implications for higher education, highlighting that both EI and FI are critical, modifiable factors influencing AP. Recent empirical evidence suggests that interventions aiming to simultaneously strengthen EI and enhance frustration tolerance can yield greater benefits than targeting either construct alone, as combined development better mitigates academic frustration and promotes student success (Wu et al. 2023).

Implementing structured EI development programs within university curricula can enhance students’ ability to regulate their emotions, cope with academic stress, and sustain engagement—factors that, as demonstrated in this study, directly and indirectly improve AP (Kotsou et al. 2011; Nelis et al. 2011; Pool and Qualter 2012). Such programs may include workshops on emotional awareness, self-regulation strategies, and social skills training, which can be integrated into existing courses or delivered as standalone modules.

Similarly, interventions focused on increasing frustration tolerance may help students manage academic setbacks more effectively, thereby reducing burnout and promoting persistence. Cognitive–behavioral strategies, resilience training, and stress management workshops have all been shown to improve emotional regulation and adaptive coping mechanisms (Recabarren et al. 2019). These initiatives can be tailored to address common academic challenges and equip students with practical tools for overcoming frustration and maintaining motivation.

Educational institutions should consider incorporating both EI and frustration tolerance training into student support services and orientation programs, especially for students exhibiting high FI or low EI, to maximize compensatory effects and overall well-being. Providing faculty and staff with resources to recognize and address signs of burnout and disengagement can also help create a more supportive academic environment (Mehler et al. 2024; Powell et al. 2024; Robertson et al. 2018). These efforts may include early identification of at-risk students, peer support groups, and ongoing professional development for teaching staff.

Ultimately, the results underscore the need for a dual focus in academic success initiatives: promoting student engagement while simultaneously addressing and mitigating burnout. By fostering both EI and frustration tolerance, universities can strengthen students’ well-being and enhance their potential for academic achievement.

These practical implications align with international educational strategies emphasizing emotional and motivational competencies as key levers for student success in diverse global contexts (e.g., MacCann et al. 2019; OECD 2018).

### 4.3. Limitations and Future Directions

This study has several limitations that should be considered when interpreting the results. First, the sample was limited to students from a single institution, which may restrict the generalizability of the findings to other university populations or educational contexts. Notably, the sample exhibited a predominance of health sciences students and those in early academic years, which may bias the results toward the characteristics of this subgroup and limit applicability to students from other disciplines or more advanced courses. Future research should include more diverse and balanced samples across multiple institutions, disciplines, academic years, and cultural backgrounds to enhance external validity.

Second, the use of self-report measures for EI, FI, burnout, and engagement may introduce response bias and limit the objectivity of the results. Incorporating objective assessments—such as skill-based tests of EI (e.g., MSCEIT)—and triangulating data with teacher or peer evaluations could provide a more comprehensive understanding of these constructs and their relationship with AP.

Third, cultural factors may influence the expression and role of EI, FI, and engagement. Emotional expression, tolerance for frustration, and the meaning of academic success can vary significantly across cultural contexts. Future studies should explore cross-cultural differences to determine the extent to which these findings can be generalized beyond the current sample.

Fourth, the cross-sectional design of this study precludes any conclusions about causality. Longitudinal research is needed to clarify the directionality of the relationships among EI, FI, burnout, engagement, and AP. Experimental studies that manipulate EI or FI through targeted interventions could also help establish causal links and evaluate the effectiveness of such programs in improving AP.

Finally, while GPA served as the primary indicator of AP, this measure may oversimplify the complexity of academic achievement. Future research could benefit from using additional indicators, such as standardized test scores, performance-based assessments, teacher evaluations, and peer reviews, to obtain a more nuanced and comprehensive picture of AP.

In addition, future studies should examine the relationships between academic resilience and other non-cognitive variables (e.g., academic self-concept, self-efficacy, causal attributions) that may contribute to AP (Miñano and Castejón 2011). By addressing these limitations, future research can provide deeper insights into the mechanisms linking emotional and motivational factors to AP and inform the development of more effective educational interventions.

## 5. Conclusions

This study demonstrates the significant impact of EI and FI on university students’ AP. The results indicate that enhancing EI serves as a valuable resource for fostering engagement and reducing burnout, thereby positively contributing to AP. Conversely, managing FI can also improve academic outcomes, as it reduces burnout and enhances engagement. The findings underscore the importance of addressing both EI and FI in educational settings to promote student well-being and academic success.

These findings highlight the critical role of addressing both EI and FI within educational settings to promote student well-being and academic success. Moreover, interventions targeting these factors have potential benefits for students across diverse countries and academic environments, reflecting the global relevance of emotional and motivational challenges in higher education.

Thus, this integrated approach holds promise for informing international educational policies and practices aimed at enhancing academic outcomes through emotional and motivational resource management.

## Figures and Tables

**Table 1:** Mean, standard deviations, Cronbach’s Alpha (on the diagonal), and correlations among variables/dimensions.

Variables/Dimensions	M	SD	1	2	3	4	5	6	7	8	9	10	11	12	13	14
1. AP	5.34	0.67	—													
2. EI/Attention	1.70	0.67	−.04	0.91												
3. EI/Clarity	1.67	0.67	.31 ***	.24 ***	0.92											
4. EI/Repair	1.78	0.69	.32 ***	.14 ***	.56 ***	0.90										
5. E/Vigour	2.35	0.76	.34 ***	.12 ***	.31 ***	.36 ***	0.86									
6. E/Absorption	2.64	0.66	.27 ***	.15 ***	.25 ***	.29 ***	.63 ***	0.89								
7. E/Dedication	2.51	0.69	.32 ***	.10 *	.29 ***	.33 ***	.72 ***	.72 ***	0.88							
8. B/Emotional exhaustion	2.40	0.77	−.36 ***	.11 **	−.32 ***	−.34 ***	-.28 ***	−.24 ***	−.28 ***	0.91						
9. B/Cynicism	2.02	0.87	−.26 ***	.06	−.20 ***	−.23 ***	−.21 ***	−.24 ***	−.22 ***	.51 ***	0.72					
10. B/Academic self-efficacy	2.21	0.83	−.29 ***	.02	−.33 ***	−.38 ***	−.38 ***	−.36 ***	−.37 ***	.58 ***	.59 ***	0.79				
11. FI/Discomfort intolerance	1.89	0.62	−.29 ***	.14 ***	−.24 ***	−.28 ***	−.24 ***	−.27 ***	−.27 ***	.37 ***	.29 ***	.29 ***	0.85			
12. FI/Entitlement	1.94	0.65	−.25 ***	.12 ***	−.25 ***	−.25 ***	−.19 ***	−.22 ***	−.22 ***	.33 ***	.27 ***	.27 ***	.64 ***	0.86		
13. FI/Emotional intolerance	2.01	0.66	−.28 ***	.16 ***	−.30 ***	−.27 ***	−.23 ***	−.26 ***	−.24 ***	.36 ***	.28 ***	.25 ***	.65 ***	.63 ***	0.87	
14. FI/Achievement	2.11	0.62	−.23 ***	.23 ***	−.19 ***	−.21 ***	−.13 **	−.12 **	.08*	.31 ***	.20 ***	.19 ***	.60 ***	.60 ***	.66	0.82

Note. * *p* < .05, ** *p* < .01, *** *p* < .001. AP: Academic Performance; EI: Emotional Intelligence; E: Engagement; B: Burnout; FI: Frustration intolerance.

**Table 2:** Global fit of structural model.

Construct Criteria	Measures of Goodness of Fit (SEM)				
	c2/df (<5)	CFI (≈1)	TLI (≈1)	RMSEA (≈0)	SRMR (≈0)
	2.93	0.969	0.958	0.055	0.044

Note: χ^2^/df: chi-square–degrees of freedom; CFI: Comparative Fix Index; TLI: Tucker–Lewis Index; RMSEA: Root Mean Square Error of Approximation; SRMR: Standardized Root Mean Square Residual.

**Table 3:** Estimated regression coefficients.

					β 95% Confidence Intervals				
Predictor Variable		Response Variable	Estimate	S.E.	β	Lower	Upper	z	*p*
EI	→	E	0.548	0.0671	0.472	0.379	0.566	8.16	<.001
EI	→	B	0.539	0.0738	−0.472	−0.580	−0.364	−7.31	<.001
EI	→	AP	0.266	0.0920	0.209	0.072	0.346	2.89	.004
FI	→	B	0.333	0.0670	0.276	0.170	0.038	4.98	<.001
FI	→	E	−0.149	0.0574	−0.121	−0.211	−0.031	−2.59	.010
FI	→	AP	−0.166	0.0658	−0.123	−0.217	−0.030	−2.53	.012
E	→	AP	0.168	0.0510	0.154	0.063	0.255	3.31	<.001
B	→	AP	−0.174	0.0659	−0.157	−0.270	−0.043	−2.65	.008

Note: Standard Error (SE), Academic Performance (AP), Engagement (E), Burnout (B), Emotional Intelligence (EI), Frustration Intolerance (FI).

## Data Availability

The data are not publicly available due to privacy and ethical restrictions. However, anonymized datasets may be provided by the corresponding author upon reasonable request.
